# Detecting wing fractures in chickens using deep learning, photographs and computed tomography scanning^☆^

**DOI:** 10.1016/j.psj.2025.105264

**Published:** 2025-05-05

**Authors:** Kacper Libera, Dirk Schut, Effrosyni Kritsi, Louis van Steijn, Timothy Dallman, Len Lipman

**Affiliations:** a Institute for Risk Assessment Sciences (IRAS), Utrecht University, Yalelaan 2, 3584 CM Utrecht, the Netherlands; b Computational Imaging Group, Centrum Wiskunde & Informatica (CWI), Science Park 123, 1098 XG Amsterdam, the Netherlands; c Meyn Food Processing Technology B.V., Westeinde 6 1511MA, Oostzaan, the Netherlands

**Keywords:** Food inspection, Animal welfare monitoring, X-ray inspection, Artificial intelligence, CT

## Abstract

Animal welfare monitoring is a key part of veterinary surveillance in every poultry slaughterhouse. Among the animal welfare indicators routinely inspected, the prevalence of wing fractures and soft tissues injuries (e.g. bruises) is particularly relevant, because it is related to acute pain and suffering in injured birds. According to current practice, assessment corresponds to visual examination by animal welfare officers. However, taking into consideration the speed of the production line and limitations associated with human inspection (e.g. different visual perception, subjectivism and fatigue), new more objective and automated techniques are desirable. Therefore, the aim of this study was to assess the applicability of three deep learning classification models to detect fractures and/or bruises based on computed tomography (CT) scans and photographs of the wings. Namely, 1. Model_CT (two categories: 1.BROKEN and 2.NON_BROKEN) detecting fractures based on CT scans, 2.Model_Photo_Fractures (1.FRACTURES and 2.NO_FRACTURES) detecting fractures based on photographs and 3.Model_Photo_Bruises (1.BRUISES and 2.NO_BRUISES) detecting bruises based on photographs. To train, validate and test these models 306 CT scans and 285 photographs were collected. The 3D ResNet34 and 2D EfficientNetV2_s architectures were used for the CT and Photo_Models, respectively. The models reached an accuracy of 98 % (Model_CT), 96 % (Model_Photo_Fractures) and 82 % (Model_Photo_Bruises). All in all, applying deep learning to the combination of CT scanning and photography can help to objectively recognize wing fractures and bruises. Consequently, it might lead to more accurate and objective animal welfare monitoring and ultimately to raised animal welfare standards.

## Introduction

Veterinary inspection is a crucial part of the workflow in every slaughterhouse in the European Union (**EU**). It is not only oriented towards ensuring a high level of safety and quality of the final product (meat), but also focuses on other important aspects of animal production such as animal welfare monitoring (Regulation of European Commission (EC) No 853/2004; Regulation (EU) 2017/625; Council Regulation (EC) No 1099/2009). There are different ways in which animal welfare can be measured in the slaughterhouse. A common and practical approach is to use criteria that can be quantified and ideally objectively measured. These are referred to as *animal welfare indicators*. There are different animal welfare indicators described among farm animals intended for slaughter. For example, the extent of fecal contamination (cleanliness) of the hide in cattle can provide information on the quality of bedding and housing in general of animals being slaughtered (Hauge et al., 2012). Whereas scratches and/or bruises on the pig skin can reflect the level of aggression/fighting during loading and transportation and/or human handling during these activities (Barington et al., 2016).

Poultry, in particular broiler chickens, stand out from the other farm animals due to their relatively small size and weight and that they are often kept in flock exceeding thousands of animals. Chickens need to be placed in special crates before transportation and this procedure is often done manually by specialized catching teams, who are not always handling them with care (Kittelsen et al., 2018). These characteristics make the chickens extremely prone to severe injuries including wing fractures and dislocations, which are painful and impact animal welfare (Gentle, 2011). Therefore, the prevalence of chicken carcasses with broken/injured wings is an important animal welfare indicator monitored at the slaughterhouse.

According to Regulation 1099/2009 (Council Regulation (EC) No 1099/2009) it is required that in every high scale producing poultry slaughterhouse (more than 150 000 birds slaughtered per year) there must be a dedicated employee (animal welfare officer), who takes responsibility in animal welfare monitoring in various stages of the processing at the slaughterhouse. The animal welfare officer`s tasks include manual (human) inspection, which is mostly visual inspection, usually performed on a pre-selected part of the flock meant to be representative for the whole batch of chickens. However, it is important to note that modern poultry slaughterhouses are capable of processing hundred-thousands of birds daily, often with a production line speed exceeding more than 10 000 birds per hour (Pacholewicz et al., 2019).

Thus, the reliability of human inspection is questionable due to hectic characteristics of this production type. Furthermore, it has been proven that the subjectivism, fatigue and monotony negatively affect the accuracy of any human inspection (Arzoomand et al., 2019; EFSA opinion, 2012).

Imaging methods, which are non-destructive testing techniques, are an attractive potential alternative to human inspection, because they may provide a higher inspection speed and consistency at a lower cost. One technique with proven accuracy in detecting injuries is X-Ray Computed Tomography (**CT**) scanning. It is widely used in human and veterinary diagnostics (Greco et al., 2023; Withers et al., 2021). Briefly, X-Rays pass through the analyzed object at multiple angles, typically over 360 degrees. The variations in density across different tissues, such as muscle and bone, alter the intensity of the X-rays that pass through the object and reach the detector, resulting in a 2D projection image at each angle. These projections are then processed to generate a 3D dataset/volume allowing for the creation of sequential images, or slices, of the object's internal structure (Hermena and Young, 2022).

The key advantage of CT scanning is its visualization of internal structures of the object in a non-invasive way. This method is very accurate and useful in detecting hard tissue injuries – bone fractures and joints dislocations (luxations), which are type of wing injuries that are commonly observed in poultry slaughterhouses (Libera et al., 2024).

However, not all types of injuries can be detected using CT scanning. In particular soft tissue injuries including bruises, petechiaes and haematomas are practically impossible to be recognized in CT scans (Libera et al., 2024). Nevertheless, soft tissue injuries can be easily revealed using a visible light camera, because these lesions usually have different coloration (red/purple/pink) than the intact parts of the carcasses. Thus, CT scanning needs to be combined with photography to cover the whole variety of possible injuries encountered in practice. Photography systems can also detect other abnormalities when examining chicken carcasses for example fecal contamination (Sandberg et al., 2023).

The shared benefit of these two techniques (CT scanning + photography) is that analysis and interpretation can be automated using artificial intelligence (**AI**) techniques, for example deep learning and in particular convolutional neural networks (**CNNs**). CNNs are specialized in image analysis and classification and have already been investigated in studies with animal welfare monitoring context including camera systems used during the primary production of broilers (Peña Fernández et al., 2018) or in pigs (Matthews et al., 2017). In CT scans the application of CNNs has been also described in companion animal studies including kidney volume determination in dogs (Ji et al., 2022) or kidney calculi (Ji et al., 2023). However, studies focusing on detecting injuries are only found in human medicine for recognizing different types of fractures (Dankelman et al., 2023).

Therefore, the aim of our study is to create and evaluate the performance of a CNN models, which are recognizing injured chicken wings based on their CT scan or photography. This solution can potentially lead to more reliable animal welfare monitoring and higher profitability of the poultry processing by reduced inspection costs. The proposed method of animal welfare monitoring in the slaughterhouse would put extra pressure and attention to careful animal handling before slaughter, because misconduct would be easily detected.

## Materials and methods

### Sample collection and study design

**Fig. 1:**
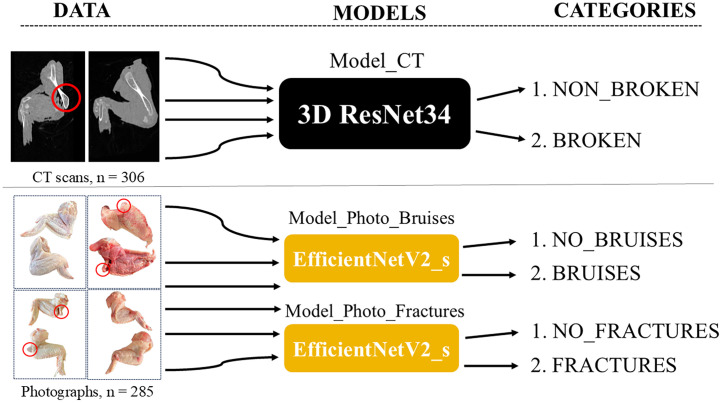
A visual explanation of the study design.

In total 306 chicken wings (Ross 308) have been collected from the slaughterhouse located in the Netherlands, which uses a CO_2_ chamber for stunning and an automatic system for bleeding. The samples were collected during 5 sampling days from July 2023 to May 2024. The wing typically consisted of humerus, radius, ulna, carpus, and manus bones with surrounding musculature and skin commercially, referred to as drumette, wingette, and tip. The samples were transported from the slaughterhouse to the laboratory in the cooling box (<4°C). All 306 wing samples were CT scanned, and 285 of them were photographed. Due to a human mistake 21 wings (2 batches) were not photographed before discarding. The brief explanation of study design is given in Fig. 1.

### CT Scanning and CT Scans Processing

All samples were scanned using a SOMATOM Definition AS CT scanner (Siemens Healthineers, the Hague, the Netherlands) at the veterinary hospital of Utrecht University, the Netherlands. The scanner's software presets were utilized to suggest the optimal combination of voltage and current based on sample size, thickness, and density. In most cases, the parameters were set to 120 or 80 kV and 80 to 150 mA, slice thickness = 0.6 mm. The samples were scanned either on the same day (as they were slaughtered) or the following day depending on the availability of the CT scanner. For time-efficiency reasons the wings were not scanned individually but were scanned in randomly allocated batches of 6-12 wings. In total, 30 batches were scanned.

The CT scans were saved as DICOM files. Since every DICOM file represented a batch (including 6-12 wings), the individual wings needed to be extracted and cropped. All the wings (*n* = 306) have been cropped manually using 3D Slicer (version 5.6.2) and saved as a separate file using the *.nii.gz file extension.

### Photographing and photographs processing

All the samples have been photographed from both sides (iPhone SE 2020 camera with 12 megapixels, resolution 3,024 × 4,032 pixels). During the first two days of photographing the samples were placed directly on the laboratory table and manually flipped to capture both side using natural (daylight) light exposure only. Whereas, during the other 3 days of photographing the samples were put in a black box before taking a photograph. A selfie ring light (650 lumen, daylight 6500 K) was used as an artificial light source to keep similar lightning conditions on different experimental days and consequently improve the methodology.

During photography processing the background was removed manually in all photographs using Paint 3D (Microsoft, Redmond, the United States). In the next step the photographs of both sides were merged into one photograph (see Fig. 1) and saved as *.png file with uniform resolution set to 394 × 680 pixels (height x width).

### Sample classification – ground truth labels and models development

For clarity, unique category names (labels) were created for each model. All the CT scans were categorized by two veterinarians into one of two categories (categories: **NON_BROKEN** and **BROKEN**). Similarly, all the photographs were categorized by the same two veterinarians into 2 categories, however it was conducted in 2 rounds, because two different ways of categorization were applied. In the first round the veterinarians were categorizing photographs based on the presence of bruises (categories: **NO_BRUISES** and **BRUISES**). While in the second round the presence of a fracture was the classification criterion (categories: **NO_FRACTURES** and **FRACTURES**). The overview of the classification criteria can be found in Table 1. In the current study three deep learning models were developed:

1. model to detect wing fractures based on CT scans (**Model_CT**).
2. model to detect soft tissue injuries (bruises) based on photographs (**Model_Photo_Bruises**).
3. model to detect wing fractures based on photographs (**Model_Photo_Fractures**).

**Table 1:** Criteria for data labeling for three deep learning models (1. based on CT scans for fracture detection, Model_CT and 2. based on photographs for bruises detection, Model_Photo_Bruises and 3. based on photographs for fracture detection, Model_Photo_Fractures).

	Category	
Model_CT	NON_BROKEN	BROKEN

	• no visible signs of fractures or dislocations in CT scan	• presence of fracture in any of: humerus, ulna, radius or bones of manus visible in CT scan OR • presence of elbow or carpus dislocation visible in CT scan
Model_Photo_Bruises	NO_BRUISES	BRUISES
	• no visible soft tissue injuries (bruises or hemorrhages)	• visible soft tissue injuries (bruises or hemorrhages)
	• fracture and/or dislocation might be present	
Model_Photo_Fractures	NO_FRACTURES	FRACTURES
	• no visible fracture or dislocation	• visible fracture or dislocation
	• soft tissue injuries (bruises or hemorrhages) might be present	

CT scan, Computed Tomography scan; Model_CT, model to detect wing fractures based on CT scans (binary classification); Model_Photo_Fractures, model to detect wing fractures using photographs (binary classification); Model_Photo_Bruises, model to detect soft tissues injuries (bruises) using photographs (binary classification).

### Train, validation, test dataset split for Model_CT, Model_Photo_Bruises and Model_Photo_Fractures

The whole CT scan dataset (*n* = 306) and photograph dataset (*n* = 285) were randomly split into train, validation and test sets with a ratio: 0.6: 0.2: 0.2, respectively. Stratified sampling was used on the label and on the day the image was acquired. The train sets were used for training the neural networks, the validation sets were used for early stopping and hyperparameter optimization, and the test sets were used for evaluating the performance. Table 2 provides an overview about the train, validation and test datasets for different models.

**Table 2:** Overview of the number of samples in training, validation and test sets for classification model based on CT scans (Model_CT) and two models based on photographs (Model_Photo_Bruises and Model_Photo_Fractures).

	Training set	Validation set	Test set	Total	Class balance
Model_CT					
NON_BROKEN	104	34	34	172	NON_BROKEN: BROKEN
BROKEN	82	26	26	134	0.56: 044
			Sum:	306	
Model_Photo_Bruises					
NO_BRUISES	94	31	31	156	NO_BRUISES: BRUISES
BRUISES	81	24	24	129	0.55: 0.45
			Sum:	285	
Model_Photo_Fractures					
NO_FRACTURES	86	27	27	140	NO_FRACTURES: FRACTURES
FRACTURES	89	28	28	145	0.49: 0.51
			Sum:	285	

Model_CT, model to detect wing fractures based on CT scans (binary classification); Model_Photo_Fractures, model to detect wing fractures using photographs (binary classification); Model_Photo_Bruises, model to detect soft tissues injuries (bruises) using photographs (binary classification).

### X-Ray CT scanning model (Model_CT)

The network architecture was a 3D ResNet34. It was converted from a 2D ResNet3D using the timm_3D library (Solovyev et al., 2022). The 2D version of the network had been pretrained on Imagenet1k, and these weights were also converted to 3D by repeating the 2D weights along the depth dimension and dividing by the filter depth. Moreover, the weights of the original three input channels (RGB-color) were summed to obtain one grayscale input channel. 1000 Hounsfield units were added to the CT values so air was mapped to zero, and the CT values were divided by the standard deviation of the foreground voxels (those with a Hounsfield value of more than −200) to match the scale of the pretrained weights. Data augmentation was applied to prevent overfitting on individual scans. Chicken wings naturally vary in size and shape, so geometric transformations over roughly the same range were performed as augmentation to increase the data set size without causing a large shift in the distribution of the training data. Moreover, random noise was added, similar to the measurement noise present in CT reconstructions. The augmentations were implemented using the TorchIO library (Perez-Garcia et al., 2021). The augmentations consisted of flipping along each axis, 3 mm elastic deformations based on a 7 × 7 × 7 grid, 15 % non-uniform scaling along each axis, 360 degree random rotations along each axis, and additive Gaussian noise with a standard deviation of 10 Hounsfield units. The flipping, elastic deformation, affine (scaling and rotation), additive noise, augmentations were applied with a 100 %, 5 %, 90 %, and 50 % probability respectively.

The cost function was the binary cross entropy (**BCE**) and the optimizer was ADAM. A batch size 7 was used on 4 GPUs, resulting in an effective batch size of 28, which is in the range recommended by recent research (Lin, 2022; Kandel and Castelli, 2020). The training duration was 10000 epochs (3.5 days on 4 Titan X GPUs), but early stopping was applied to prevent overfitting. At the end of every epoch, the validation set BCE was calculated, and the version of the network with the lowest validation set BCE was used in the results section.

### Models based on photographs (Model_Photo_Fractures and Model_Photo_Bruises)

The network architecture was an EfficientNetV2_s (Tan and Le, 2021) from Torchvision (Marcel and Rodriguez, 2010). The cost function was the BCE and the optimizer was ADAMW. The network was pretrained on Imagenet1k, and the photographs were normalized based on the weights used for Imagenet1k. Data augmentation was applied to prevent overfitting on individual images and on the changes in the lighting conditions of the scanning days. The augmentations consisted of geometric transformations, similar to the biological variation in size and shape; color transformations, similar to the changes in lighting; and random noise, similar to measurement noise. The augmentations were implemented using the Albumentations library (Buslaev et al., 2020). The augmentations were flipping along each axis, elastic deformations, rotations, shearing, non-uniform scaling along each axis, color jitter (brightness, saturation, contrast and hue) and additive Gaussian noise.

A hyperparameter optimization was performed to optimize the data augmentation parameters and the learning rate and weight decay of the optimizer. One neural network was trained for each run. Early stopping was applied to prevent overfitting and to speed up the hyperparameter optimization. The training of one run was stopped when no improvement in validation set BCE was observed for 100 epochs, and the lowest validation set BCE was used as the score of that run. A batch size 9 was used on 4 GPUs, resulting in an effective batch size of 36, which is in the range recommended by recent research (Kandel and Castelli, 2020; Lin, 2022). The network with the lowest validation set BCE was used in the results section. Separate hyperparameter optimizations were performed for detecting fractures and for detecting soft-tissue damage. Both used 250 runs of the Tree-structured Parzen Estimator as implemented in the Optuna framework (Akiba et al., 2019). The Tree-structured Parzen Estimator is the default choice in Optuna, and the recommended algorithm for a budget of 250 runs according to the Optuna documentation. In total the hyperparameter optimization took approximately two weeks on 4 Titan X GPUs.

### Performance evaluation

Several evaluation metrics have been used to assess all models` performance on the test data set including:

$$\text{Accuracy}\;=\;\frac{TP+TN}{TP+TN+FP+FN}$$

$$\text{Precision}\;=\frac{TP}{TP+FP}$$

$$\text{Recall}\;=\;\frac{TP}{TP+FN}$$

$$\text{False}\;\text{Positive}\;\text{Rate}\;\left(\mathrm{FPR}\right)\;=\;\frac{FP}{FP+TN}$$

True positive, (**TP**); a sample, which was correctly predicted as BROKEN (Model_CT), FRACTURES (Model_Photo_Fractures) or BRUISES (Model_Photo_Bruises).

True negative, (**TN**); a sample, which was correctly predicted as NON_BROKEN (Model_CT), NO_FRACTURES (Model_Photo_Fractures) or NO_BRUISES (Model_Photo_Bruises).

False positive, (**FP**); a sample, which was incorrectly predicted as BROKEN (Model_CT), FRACTURES (Model_Photo_Fractures) or BRUISES (Model_Photo_Bruises), but in fact it was NON_BROKEN (Model_CT), NO_FRACTURES (Model_Photo_Fractures) or NO_BRUISES (Model_Photo_Bruises).

False negative, (**FN**); a sample, which was incorrectly predicted as NON_BROKEN (Model_CT), NO_FRACTURES (Model_Photo_Fractures) or NO_BRUISES (Model_Photo_Bruises), but in fact it was BROKEN (Model_CT), FRACTURES (Model_Photo_Fractures) or BRUISES (Model_Photo_Bruises).

Area under Curve (**AUC**) = area underneath the **ROC** curve (receiver operating characteristic curve plotting Recall vs. FPR).

The class BROKEN/FRACTURES/BRUISES was considered as a positive class (1), while NON_BROKEN/NO_FRACTURES/NO_BRUISES as a negative (0).

## Results

### Model_CT performance on test dataset

Model_CT classified all samples correctly in the training and validation data set (accuracy = 1.0) . In the test data set, one sample was misclassified resulting in an accuracy of 0.9833. Model_CT was trained for 9558 epochs, before being stopped by the early stopping. The detailed performance of Model_CT on the test dataset is given in Table 3.

**Table 3:** Performance on the test dataset (*n* = 60) of binary classification model based on chicken wing CT scan (Model_CT).

	Accuracy	Precision	Recall	FPR	AUC
Model_CT	0.9833	0.9630	1.000	0.0294	1.0

Model_CT, model to detect wing fractures based on CT scans (binary classification); FPR, false positive ratio; AUC, area under the curve.

**Fig. 2:**
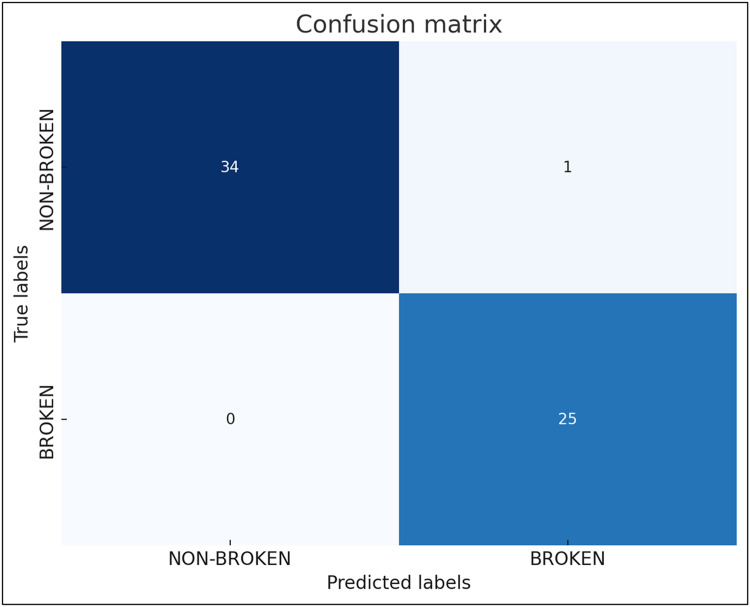
Confusion matrix based on prediction of Model_CT performed on test dataset (*n* = 60).

The exact results of the model prediction can be found in the confusion matrix (Fig. 2). The test set consisted of 60 samples.

### Model_Photo_Fractures and Model_Photo_Bruises performance on the test dataset

The performance of Model_Photo_Fractures and Model_Photo_Bruises is given in Table 4. Model_Photo_Fractures outperformed Model_Photo_Bruises in all evaluation metrics. The best performing versions of Model_Photo_Fractures and Model_Photo_Bruises were trained for 490 and 335 epochs respectively, before being stopped by the early stopping.

**Table 4:** Performance on the test dataset (*n* = 55) of binary classification models based on chicken wing photography (Model_Photo_Bruises and Model_Photo_Fractures).

	Accuracy	Precision	Recall	FPR	AUC
Model_Photo_Bruises	0.8182	0.8889	0.6667	0.0645	0.9032
Model_Photo_Fractures	0.9636	1.000	0.9286	0.000	0.9867

Model_Photo_Fractures, model to detect wing fractures using photographs (binary classification); Model_Photo_Bruises, model to detect soft tissues injuries (bruises) using photographs (binary classification); FPR, false positive ratio; AUC, area under the curve.

### Explainable AI – Grad-CAM

**Fig. 3:**
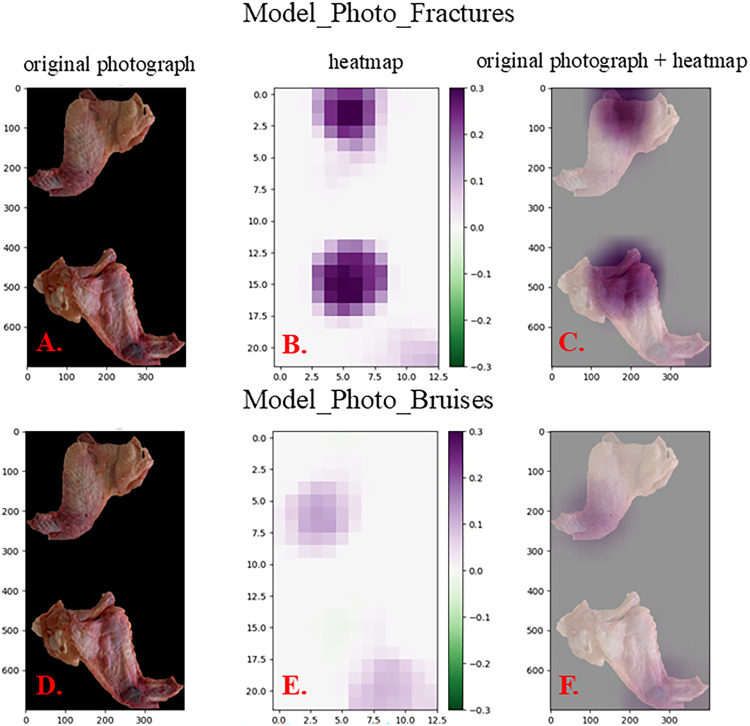
The photograph of a sample correctly classified as FRACTURES (Model_Photo_Fractures, panels **A.-C.**) or BRUISES (Model_Photo_Bruise, panels **D.-F.**) with a corresponding heatmap created by Grad-CAM. The purple regions of the heatmap highlight important parts of the image for the classification (decision-making) made by Model_Photo_Fractures and Model_Photo_Bruises. The photograph of the same sample was intentionally used to present different parts of the image important for the classifications made by the models.

To provide a visual explanation of the models’ predictions the Grad-CAM method was used (Selvaraju et al., 2017). An example of the original photograph with the corresponding heatmap is given in Fig. 3 (Model_Photo_Fractures and Model_Photo_Bruises). Since these two models were trained on the same dataset, the same sample was presented to highlight the difference between the decision-making processes of the models during detecting fractures or bruises (Model_Photo_Fractures and Model_Photo_Bruises, respectively). The protruding humerus and elbow are highlighted (in purple) in panels B.-C. (Model_Photo_Fractures), while a hematoma located in the area of carpus is highlighted in panels E.-F (Model_Photo_Bruises) of the Fig. 3.

**Fig. 4:**
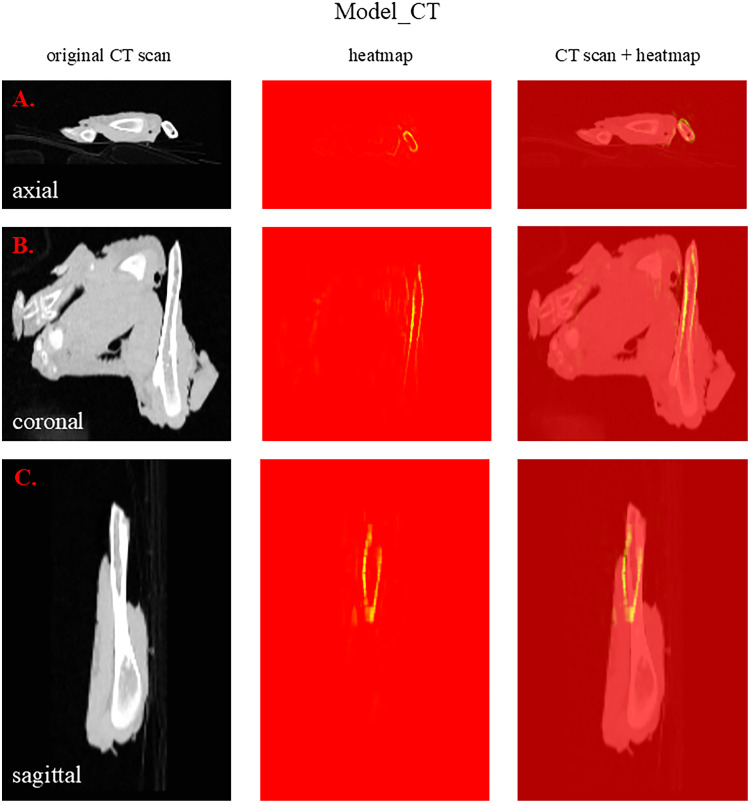
An example of a CT scan correctly classified as BROKEN by Model_CT with corresponding heatmap created by guided Grad-CAM. The same sample is shown in three planes: axial (A.), coronal (B.) and sagittal (C.). The yellow-green regions of the heatmap highlight the important parts of the image for the classification (decision-making) made by Model_CT.

Fig. 4 presents a CT scan with a corresponding heatmap in different planes (A.-axial, B.-coronal and C. sagittal). The protruding humerus is highlighted in yellow-green in all three planes. Due to the low resolution of the final convolution layer of the 3D ResNet34 model, the Grad-CAM heatmap had a very low resolution. Therefore, for this model a guided Grad-CAM heatmap is shown instead.

## Discussion

The aim of this study was to develop CNN models to identify injured wings based on CT scans and photographs in the context of animal welfare monitoring. Gathering the information on prevalence of the carcasses with broken wings is crucial for objective assessment of animal handling related to animal welfare shortly before slaughter (e.g. loading, transportation and unloading). Furthermore, wings are also valuable cuts of the chicken carcass and any detectable injury excludes them from being fit for human consumption in many countries, so detecting wing injuries is also important for the food business operator.

Our classification models reached 98 %, 96 % and 82 % of accuracy for Model_CT, Model_Photo_Fractures and Model_Photo_Bruises, respectively. It ensures an accurate and objective way of inspection, creating opportunities for improved and automated veterinary inspection of chicken wings in the slaughterhouse.

### Wing fractures as an animal welfare indicator

The prevalence of wing fractures has been proven to be a reliable tool to assess the animal handling during loading, transportation and unloading/hanging. Theoretically wing fractures can occur at the primary production stage, but according to Kittelsen et al. (2015) most of the wing fractures occur during evacuation of transport containers and shackling, so during pre-slaughter handling in the slaughterhouse. The prevalence of wing fractures varies between 0.0138 % and 5.7 % (Cockram et al., 2020; Valkova et al., 2021). However, when interpreting the wing fracture prevalence it is important to determine which method of stunning was used in the slaughterhouse, because there are some differences in prevalence of wing fractures described between different methods of stunning. In the research conducted by Riggs et al. (2023) there was a significantly higher prevalence of wing fractures in chickens stunned in controlled atmosphere stunning comparing to electrical stunning (3.6 % vs 2.2 %, respectively). The difference can probably be explained by the excess of wings flapping during controlled atmosphere stunning leading to an increased number of injuries observed. In some countries there are very specific thresholds above which the catching team, farmer, or slaughterhouse can receive a fine from the food safety/animal welfare authority. For example, in the Netherlands the acceptable threshold for wing fracture prevalence is 1 % for the flock since the beginning of 2024 (Dutch Food Safety Authority Official Website; in Dutch). In 2023 it was 2 %, so there is a noticeable trend to raise the animal welfare standards (Dutch Food Safety Authority Official Website; in Dutch).

The key difficulties when inspecting carcasses for wing fractures are the number of chickens slaughtered (e.g. size of the batch) and the speed of the line often exceeding 3 chickens per second (Pacholewicz et al., 2019). Usually only a moderate part (e.g. couple of hundreds) of the slaughtered batch (e.g. several thousands) are being checked (Dutch Product Boards for Livestock, Meat and Eggs, 2021), which also raises doubts about the sampling and inspection strategy being representative. Moreover, it is in contrary to post-mortem inspection, when every carcass needs to be checked individually (Regulation (EU) 2017/625). Finally, the clear definition of the injury/fracture among different inspectors might also be questionable. Therefore, new and more objective techniques, as described in this study, are highly desired.

### Automated wing injury detection

The modern poultry meat industry is characterized by extremely high efficiency, rapid speed of the line and mechanization/robotization at almost every stage of the processing (FAO, 2024). We believe that animal welfare inspection (or food inspection in general sense) should also be modernized. In the current study we examined two imaging techniques, which can be automated with artificial intelligence methods. Based on our previous research we know that combination of CT scanning and photographs has a synergistic effect (Libera et al., 2024). In our desired setup in the slaughterhouse, all the carcasses would be first examined with camera and classified by two models in one out of 2 categories. In the next step all the carcasses are CT scanned. If the carcass would be categorized by Model_Photo_Fractures or Model_Photo_Bruises as injured (cat. BRUISES and/or cat. FRACTURES) then CT scanning would be performed and act as a confirmation of hard tissue injury (dislocation/fracture). In other words, if there is only soft tissue injury (bruises/petechias/hematomas etc.) Model_Photo_Bruise will detect it, but CT scanning would exclude bone/joints injury by classifying it as NON_BROKEN. Alternatively, if models based on photographs would classify wing as intact (cat. NO_BRUISES and/or cat. NO_FRACTURES) and Model_CT classifies it as BROKEN, then it would be considered as fracture, which occurred after bleeding (post-killing fracture), so it would not be considered as an animal welfare issue. Nevertheless, it is still important information for the food business operator, because these wings cannot be used for human consumption (Regulation (EU) 2017/625). Obviously, there might be a discrepancy between the output of Model_Photo_Fracture and Model_CT because they work independently based on different modalities. For example, Model_Photo_Fracture can classify a sample as NO_FRACTURE, but according to Model_CT the same sample is classified as BROKEN and vice versa. In these cases we claim that the output of Model_CT should be decisive, that is because CT scanning is specialized and superior in detecting fractures comparing to photographs. Alternatively, wings with mutually exclusive predictions, can be subjected to human (re)inspection by an official veterinarian.

Therefore, in the current study we propose to combine the key advantage of CT scans (recognizing fractures) with the merit of visual inspection (ability to distinguish colors) to get full insight about wing injuries that are reported in the slaughterhouse. The objectively measured number of wing fractures can be used as animal welfare indicator, and if it exceeds a given threshold food safety/animal welfare authorities can issue a fine to the catching team, transportation company or other parties involved. It can also help to compare animal welfare standards between slaughterhouses and transportation companies and ultimately raise the animal welfare level. Moreover, the number of post-killing fractures (not an animal welfare issue) can be valuable feedback to food business operators about the slaughter machinery performance.

The usefulness of CT scans in the inspection of other types of food has been already described, for example in detecting browning in apples (Wood et al., 2024; Schut et al., 2024), detecting pest in mangos (Kim et al., 2022) or analysis of coffee beans (Cloete et al., 2019). In our research we examined 306 CT scans of chicken wings and then created a deep learning model, which is able to categorize wings as BROKEN or NON_BROKEN with 98 % accuracy. This accuracy is satisfactory, taking under consideration that we have well-balanced classes (ratio NON_BROKEN:BROKEN = 0.56:0.44), so if the model would always predict NON_BROKEN it would get an accuracy of 56 %. The fact that the train and validation set accuracy is 100 % suggests that the performance of the model on the test data is limited by the amount of data. Therefore, with more data the same model may achieve an even higher accuracy. The AUC of 100 % shows that the output of the network already consists of two clusters that can be separated with the right threshold, also suggesting that further improvements on the test dataset are possible. We examined the one sample that was misclassified and it was characterized by small fractures in antebrachia bones. It is important to note that most of our BROKEN samples were identified with elbow luxation or fraction of humerus, so the model was not able to efficiently learn these kind of fractures. Nevertheless, in practice elbow luxation is one of the most prevalent types of wing injury, so technically bones are not fractured, but the elbow joint is dislocated.

Computer (visual) vision systems have been investigated and occasionally are also implemented in practice in poultry slaughterhouses (Sandberg et al., 2023). The key advantage of a camera system over CT scanning is the relatively low cost of installation and recording and no need for extra precautions (e.g. X-ray protection). In our research we decided to create neural networks based on photographs, because fractures/dislocations are not the only type of wing injuries observed in poultry slaughterhouses. There are cases, where the bones and/or joints seem intact, but the wings are covered with bruises/hematomas or are swollen, so probably only soft tissues are affected. Undoubtedly this type of injury is also painful, and thus detrimental for animal welfare. With a visible light camera (photographs) alternations of colors can be easily detected (CT scans are gray-scale images). Consequently, samples covered with bruises but with no hard tissue injury (fracture/dislocation), are also detectable (category BRUISES from Model_Photo_Bruises). Another challenge for CT scanning are samples, where a bone/joint injury does exist, but there are no other pathological changes on the wing skin, suggesting no inflammatory reaction to the damaging factor. Therefore, it can be concluded that this type of injury happens after the chickens are bled-out (dead, so not able to feel pain anymore), so it should not be considered as animal welfare issue. The most probable reasons for these kinds of injuries are faulty or suboptimal performance of the slaughterhouse machinery. For example, rubber fingers can cause mechanical damages to the carcass during defeathering (Shung et al., 2022). Thus, to identify the fractures caused by the defeathering machines (chicken pluckers) we would suggest to scan/take photographs immediately after the plucking.

Utilizing photographic imaging we observed a lower performance of the network detecting bruises comparing to fractures (82 % vs 96 %), which was in accordance with our expectations. During data labeling we observed a high variety among samples in terms of size, location, shape, color, and intensity of the bruising. Some authors have tried to establish a minimal diameter of a bruise to be counted as injury, because small reddish lesions might not always indicate an injury, but rather a contamination or local bleeding/change of the skin color. For example, in the study by van Emous et al. (2024) the threshold for wing hematomas to be considered as a bruise were lesions larger than 2 cm^2^. On the other hand, according to Dutch Product Boards for Livestock, Meat and Eggs (Dutch Product Boards for Livestock, Meat and Eggs (PVE), 2021) the lesions should be monitored and counted as bruise during animal welfare inspection if its diameter is at least 3 cm, which leads to omission of smaller lesions and consequently possible underdetection of animal welfare-relevant findings.

Lighting conditions can also influence the perception of the colors, that is why we switched from photographing in natural lighting to a controlled lighting setup after the first 2 days. Moreover, we expect that the color jitter data augmentations improved the robustness of the model to lighting variations. Nevertheless, we expect that the lighting variation in our dataset had a negative effect on the detection performance. When the same controlled lighting setup is used over the whole dataset in future experiments we expect that a better detection performance is possible. In a factory setting a controlled lighting setup should also be achievable. The definition of fractures seems less disputable so we expect fracture detection to be less sensitive to variations in lighting.

From the production point of view the accuracy should reach as high value as possible, because in real-life application (slaughterhouse) the actual observed prevalence of carcasses with fractures is estimated at several percent (up to 5 %). However, all the previous research was based on human (visual) inspection, which we believe might have led to a significant underestimation of this animal welfare indicator. Two of our models (Model_CT and Model_Photo_Fractures) reached accuracy above 95 % (98 % and 96 %, respectively), which might be a satisfactory performance for the industry. It is important to note that in human diagnostics similar accuracy levels are achieved and these results are considered as satisfactory and comparable to senior practicing radiologists (Moezzi et al., 2021). Model_Photo_Bruises performed at accuracy of 82 %, which is a satisfactory result for this research, but not yet for the industry. As discussed before, identifying bruises seems to be more challenging task for both human eyes and computer vision and currently this problem seems to be overlooked during animal welfare inspection.

However, not only accuracy should be discussed when assessing a classification model, because misclassifications (false positive or false negative) might have different meanings and consequences in the different real-life applications. In our research a false positive sample would be a wing classified as fractured (Model_CT and Model_Photo_Fractures) or injured (Model_Photo_Bruises), but in fact it was not fractured and/or injured. The false positive samples lead to overestimation of fractured/injured wings and consequently unnecessary food waste, because these wings cannot be sold for human consumption. This type of misclassification is not desired by both animal welfare authority and food business operator. Precision is a metric used to assess the quality of a positive prediction and in this study it reached 96 %, 100 % and 89 % for Model_CT, Model_Photo_Fractures and Model_Photo_Bruises, respectively. Similarly, precision above 95 % can be considered as satisfactory for both research and industry, but precision for Model_Photo_Bruise should be improved before industrial application.

On the other hand, a false negative sample would be a wing classified as healthy, but in fact it was fractured and/or injured. The false negative samples result in underestimation of fractured/injured wings, which is a significant challenge for an objective animal welfare assessment. Moreover, the consumers would not be buying or just would not be satisfied with chicken wings with visible fractures or injuries being offered for sell at retail. For the food business operator these samples might be ambiguous. On the one hand, more chicken wings (including injuries and fractures) can be sold for human consumption increasing the profitability. On the other hand, there might be quality complaints from the retail or poultry meat processing plants, which is not desired in the end. In the output from Model_CT there were no false negative samples. Whereas, there were 2 false negatives samples in the output of Model_Photo_Fractures and 8 false negative samples in the prediction by Model_Photo_Bruises.

Another thing to consider is that the way the data is split can influence a neural network’s performance, resulting in random variations. The average and variance of the performance metrics could be measured by applying cross-validation. However, cross validation multiplies the training time (e.g. by a factor 5 for 5-fold cross validation). Given the already long computation time of training the photo models because of the hyperparameter optimization (2 weeks), performing cross validation was considered too time consuming. However, we do recommend it for future studies.

Ideally, for every classification task, there should be a benchmark available, with which the performance of every new model can be compared. However, in many classification problems including ours it is practically impossible. It is extremely difficult to objectively assess the performance of the current way of inspection (human visual inspection), because there is no “golden standard” available. The identification of injured wings will be always made based on the skills, experience and thoroughness of the animal welfare inspection and/or official veterinarian. Comparing our results with research regarding image classification from different domains (e.g. medical imaging, microscopy) we consider metrics with value 90 % or more as satisfactory (Liu et al., 2022; Soni et al., 2022).

### Explainability of our deep learning models

It is desirable to understand the decision-making process of machine learning models (the model`s categorization). Among methods that can explain how deep learning models work Grad-CAM is a popular visualization technique that is useful for understanding how a convolutional neural network has been driven to make a classification decision. It creates a heatmap highlighting the regions of the image that influenced the network’s decision-making. An example of a guided Grad-CAM image for Model_CT is given in Fig. 4. Bones and joints of the wings are highlighted in yellow-green, which was expected, because we wanted to teach our model to mainly inspect hard tissues looking for fractures and/or dislocations. In Fig. 3 there are heatmaps from Model_Photo_Fractures and Model_Photo_Bruises. We decided to present the same sample processed by these two different models to showcase the different approach between them during decision making. In the heatmap from Model_Photo_Fractures the protruding bone was highlighted, while in the heatmap from Model_Photo_Bruises a quite big haematoma in the area of carpus was highlighted.

### Image classification models development

In the literature there are already reports describing CNN models for CT scan classification tasks. Most of the studies are performed in human imaging diagnostics including determining the severity of COVID-19 lung lesions (Alshazly et al., 2021 – accuracy 93.87 %) or tuberculosis prediction (Zunair et al., 2020 – accuracy 67.5 %). There are also studies focusing on fracture detection and the accuracy in recognizing fractures ranges from 69.4 % to 99.1 % (Dankelman et al., 2023). Neural network architectures are typically not designed for a specific dataset, but for a class of tasks (classification, segmentation, object detection). Therefore, most researchers use existing architectures (ResNet, InceptionV3 or DenseNet) that have achieved good results on a different dataset within the same task class. Another benefit is that the weights of these networks can be downloaded that have already been pretrained on an existing dataset, reducing the training time and sometimes improving the final performance of the network. This is the approach that we also applied in our study.

When working with CT scans there are different ways to process them and feed them into a neural network. From a technical point of view, a CT scan is a series of 2D gray-scale images (slices) stacked on top of each other. Therefore, all the stacked CT slices can be treated as one 3D object and interpreted by a 3D neural network, which is the approach used in this paper. The key benefit of this approach is that the topographical relationships between surrounding slices are kept (e.g. a bone protruding through the chicken wing skin can be seen in multiple surrounding slices), which can help the model to learn spatial patterns. Alternatively, CT scans can be used to simulate X-ray images. From the practical (industrial) point of view using only a single X-ray projection for automated image classification would be worthwhile because acquiring X-ray images is cheaper and faster than acquiring CT scans. Moreover, 2D neural networks are more common in literature and require less computation time. However, the contrast and spatial information are lower in X-ray images than in CT scans. A benefit of simulating X-ray images from a CT scan instead of acquiring X-ray images directly is that X-ray images can be acquired from any object orientation, so many unique X-ray images can simulated for neural network training from just one CT scan.

It is important to note that at the beginning of our study we planned to train only one neural network based on photographs to detect both bruises and fractures. However, eventually we decided to train two neural networks based on photographs, because during experimenting with data augmentation we noticed that some networks perform better at detecting fractures, while the others have high performance at detecting bruises and they required different data augmentation settings. Consequently, there was a trade-off between detecting fractures and detecting bruises. Furthermore, initially we also investigated if the Model_CT can distinguish between 4 classes (the presence of bruises yes/no and the presence of fracture yes/no), but the results were not satisfactory, so we decided to keep and proceed with only two categories (BROKEN and NON_BROKEN).

### Strengths, future perspectives and limitations of the study

To the best of our knowledge this is the first study using a unique combination of CT scans, photographs and artificial intelligence for image classification task. CT scanning is mainly used in medical imaging, but we show that it might also be useful in industry including food production. Another strength of our research is that we collected photographs and CT scans of almost the same set of samples (CT scans, *n* = 306; photographs, *n* = 285). In other words for every photograph there was a corresponding CT scan. It helped us to better describe and classify every sample. From a modelling point of view, we choose to use pre-trained models and fine-tune them to our specific problems. These models were trained on thousands of examples, which increases their reliability. However, it is important to note that these models are not pre-trained on CT scans (gray-scale series of images), so we need to adjust it to our own specific needs by changing 3 channels (RGB) to one channel (gray-scale).

The application of CT scans in combination with photographs might not only be limited to animal welfare monitoring. Potentially, with one whole carcass CT scanning/photography a lot of useful information might be gathered. These include health status of the carcass, presence of contamination, or some carcass measurements that might be useful for further processing and cutting. A unique feature of the whole carcass CT scanning would be to determine the health status of the internal organs before evisceration.

One of the limitations of the current study is that it was conducted outside of the poultry slaughterhouse. Wing samples were statically photographed in the lab and CT scanned in a veterinary clinic. However, the key assumption of the study was meant to be a proof of principle and to check how accurate artificial intelligence models can correctly classify isolated wings samples (after being detached from the carcass) in a controlled (laboratory) setting. After this study, the next experiment should focus on wings while being transported on a conveyor belt. That is because one of the challenges faced when testing the setup in real-life might be that birds are being hung close to each other, which means that wings can be (partially) covered. For CT scanning it should not be an issue, because X-rays can easily penetrate the carcasses, but for visible light cameras (photographs) that might be challenging. One of the solutions would be to increase the distance between birds at the slaughter line or alternatively scan/photograph wings at the later stage of the processing. For example, wings can be photographed/scanned just after detachment from the carcass – then they lie down on a flat surface not covering each other. Finally, the target experiment should be performed on the whole carcasses hanging on the slaughter line. In this case another artificial intelligence model should first detect wings in the whole carcass image (object detection task) and then another model would classify them as healthy or injured. Another limitation, is the moderate number of samples included in the study (*n* ≈ 300), however using data augmentation during training we tried to provide as high as possible variability of samples used for training. This is very common and practical approach used in machine learning research (Mumuni and Mumuni, 2022).

Investment costs, scanning time, and radiological protection might also pose a challenge for introducing CT scanning in poultry slaughterhouses. For example, based on a rough estimation the price of a new CT scanner comparable to the one used in a study can range from 100,000 to 1,000,000 USD and the cost of daily operation (primarily electricity) should not exceed several dozen USD. For instance, in a study with a CT scanner used for medical imaging, the total energy consumption for 1 year was 26 226 kWh (Heye et al., 2020). Still, it is important to note that industrial applications might be more cost-intensive. However, rapid developments in technology might overcome these limitations and lower costs in the near future.

## Conclusion

Accurate detection of wing fractures in broiler chickens based on CT scans and photographs can be automated with artificial intelligence models. It provides a more robust and more objective method of monitoring animal welfare in poultry slaughterhouses, which would e.g. create opportunities to compare different methods of birds catching and/or handling at the farm and/or slaughterhouse.

## Funding

This study was supported by a grant from Dutch Research Council (NWO), the project title: “Universal Three-dimensional Passport for process Individualization in Agriculture (UTOPIA)”; grant no: ENWSS.2018.003.

## Data and code availability

The data used for this study are available upon reasonable request.

Code is available at: https://github.com/D1rk123/ct_wing_fracture_detection/tree/main.

## Ethics statement

Since this study did not involve any living animals, no ethical statement was required. The samples were obtained from the chickens routinely slaughtered in a commercial slaughterhouse in the Netherlands between July 2023 and May 2024.

## CRediT authorship contribution statement

**Kacper Libera:** Writing – original draft, Investigation, Data curation, Writing – review & editing. **Dirk Schut:** Investigation, Data curation, Methodology, Writing – review & editing. **Effrosyni Kritsi:** Data curation, Investigation, Writing – review & editing. **Louis van Steijn:** Funding acquisition, Writing – review & editing. **Timothy Dallman:** Conceptualization, Writing – review & editing, Supervision. **Len Lipman:** Conceptualization, Funding acquisition, Writing – review & editing, Supervision.

## Declaration of competing interests

The authors declare that they have no known competing financial interests or personal relationships that could have appeared to influence the work reported in this paper.
